# Bio-Photonic Detection and Quantitative Evaluation Method for the Progression of Dental Caries Using Optical Frequency-Domain Imaging Method

**DOI:** 10.3390/s16122076

**Published:** 2016-12-06

**Authors:** Ruchire Eranga Wijesinghe, Nam Hyun Cho, Kibeom Park, Mansik Jeon, Jeehyun Kim

**Affiliations:** 1 School of Electronics Engineering, College of IT Engineering, Kyungpook National University, 80, Daehak-ro, Buk-gu, Daegu 41566, Korea; eranga@knu.ac.kr (R.E.W.); pepl10@knu.ac.kr (K.P.); jeehk@knu.ac.kr (J.K.); 2 Eaton-Peabody Laboratories, Massachusetts Eye and Ear Infirmary(MEEI), 243, Charles Street, Boston, MA 02114, USA; namhyun_cho@meei.harvard.edu; 3 Department of Otology and Laryngology, Harvard Medical School, 243, Charles Street, Boston, MA 02114, USA

**Keywords:** optical frequency domain imaging (OFDI), optical coherence tomography, dental caries, demineralization, Bio-photonic detection

## Abstract

The initial detection of dental caries is an essential biomedical requirement to barricade the progression of caries and tooth demineralization. The objective of this study is to introduce an optical frequency-domain imaging technique based quantitative evaluation method to calculate the volume and thickness of enamel residual, and a quantification method was developed to evaluate the total intensity fluctuation in depth direction owing to carious lesions, which can be favorable to identify the progression of dental caries in advance. The cross-sectional images of the *ex vivo* tooth samples were acquired using 1.3 μm spectral domain optical coherence tomography system (SD-OCT). Moreover, the advantages of the proposed method over the conventional dental inspection methods were compared to highlight the potential capability of OCT. As a consequence, the threshold parameters obtained through the developed method can be used as an efficient investigating technique for the initial detection of demineralization.

## 1. Introduction

Precise structural imaging and quantitative evaluation are critical for the diagnosis of dental caries and research to prevent the formation of initial demineralized regions [1,2]. The depth imaging of enamel, dentin, cavities, pits, and fissures with a high-resolution is particularly important for the study of anatomical and pathological changes of the dental structure [3]. Optical coherence tomography (OCT) is a rapidly advancing optical frequency-domain imaging modality, which can provide non-invasive high-resolution cross-sectional images of dental tissues and various biological tissues [4]. This near-infrared (NIR) biomedical imaging method provides images with high axial and lateral resolutions (i.e., below 8 μm and 15 μm, respectively) [5,6], and, furthermore, OCT has been widely used in different medical applications such as ophthalmology [7,8], dermatology [9], and otolaryngology [10,11]. The methods currently in use for the detection of dental caries such as radiography, microradiography, and X-rays do not provide sufficient resolution, sensitivity, and contrast compared to OCT. Radiography is the most frequently applied inspection method in dentistry with a resolution of 50 μm, which is comparatively lower than the resolution of OCT. Furthermore, radiography is not quantitative, and it is relatively difficult to apply for the initial detection of dental caries [12,13]. Microradiography is another method that can be used to analyze caries quantitatively, but it is hard to apply this method for clinical applications [14]. Other conventional diagnostic methods such as infrared (IR) imaging, dental explorer, and visual inspection are unable to provide more accurate cross-sectional images [15]. Several research groups have determined the mineral loss and the depth of enamel caries using a histology analysis method called transversal microradiography (TMR). However, due to the requirement of a thin sectioning process, applications of the method in dentistry have been scarce [16]. The main drawback of this method is that caries can be detected only at a relatively advanced stage when remineralization is no longer possible, and due to the incapability of obtaining precise quantitative measurements, it is hard to barricade the progression of caries. Thus, owing to the non-invasive and non-destructive imaging capability, and the capability of acquiring precise quantifications such as accurate thickness and volumetric measurements, OCT has gained a significant demand in the medical field as an early diagnosis method. Although OCT has been extensively used as a powerful dental imaging technique, quantitative evaluation of enamel thickness variation, depth dependent intensity fluctuation, and volumetric analysis of enamel residual has not been broadly studied for the initial diagnosis of demineralization.

Especially in dentistry, OCT has been used to produce longitudinal images of dental tissues and caries of an orientation similar to that of the B-scan ultrasound images [17,18]. In these studies, a reduction in enamel reflectivity was observed in areas of dental caries [19]. It is considered that the decrease in reflectivity during demineralization is related to the amount of mineral loss. Few studies have demonstrated that there is two to threefold increase in the scattering coefficient at a wavelength of 1.3 μm [20]. Furthermore, a polarization sensitive OCT (PS-OCT) endoscopic system using a swept source has been implemented as a compact system for dentistry application [21]. Similarly, surface demineralization can be detected using linearly polarized light and measured backscattered signal in two orthogonal axes [22]. Some other OCT techniques have been applied to diagnose dental caries as a result of changes in the optical properties of enamel after undergoing demineralization [23]. The obtained images were quantitatively evaluated by the identification of structures, dimensions, and properties [24]. Moreover, in several review reports and research studies, OCT based dental experiments were demonstrated to verify the stronger optical backscattering signals acquired from the demineralized enamel regions, oral tissue images, caries, periodontal diseases, and oral cancers [25,26]. In addition, infrared light with long wavelengths were used in OCT for clinical applications owing to the high depth penetration [27].

In this paper, we performed an initial *ex vivo* study using a 1.3 μm wavelength laser utilized spectral domain OCT (SD-OCT) system to introduce a quantitative method to calculate the thickness and volume of remaining enamel region (enamel residual). Furthermore, we developed an algorithm to analyze the total intensity fluctuation in depth direction of OCT images, which can be useful to identify the progression of initial caries. As a result, the proposed quantification can be implemented to identify the reduction of enamel region along with the progression of the demineralization. Moreover, the capability of our SD-OCT system to perform the proposed method was validated by obtaining images of *ex vivo* caries with a high resolution and a high depth penetration.

## 2. Materials and Methods 

### 2.1. Optical Frequency Domain Imaging (OFDI) Technique

**Figure 1:**
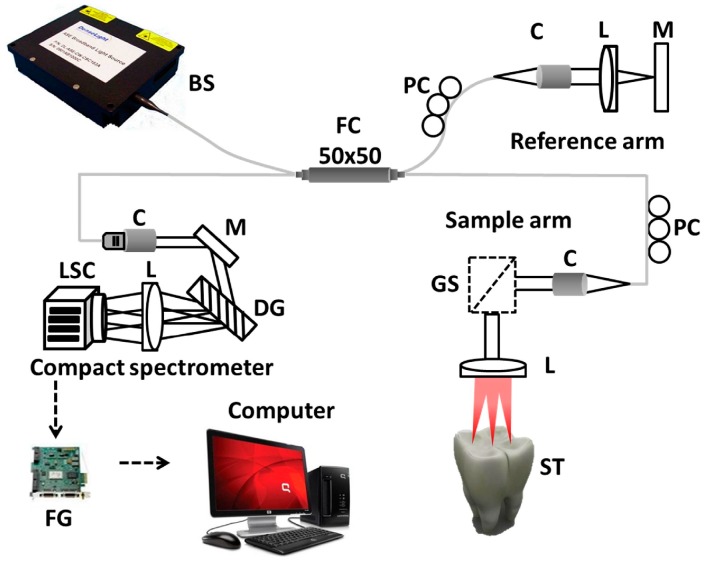
Schematic diagram of the spectral-domain optical coherence tomography (SD-OCT) system. Note the use of the following acronyms in the figure: BS: broadband source, C: collimator, DG: diffraction grating, FC: fiber coupler, FG: frame grabber, GS: galvanoscanner, L: lens, LSC: line scan camera, M: mirror, PC: polarization controller, and ST: sample tooth.

The implemented optical frequency domain imaging technique was a customized 1.3 μm SD-OCT system. The speed of the SD-OCT system was 120 frames/s when the image size was 1024 × 500 pixels, and the average output power of the system was 16 mW. In Figure 1, the broadband light source that was used for light emission is a superluminescent diode (SLED) (Denselight Semiconductors, Singapore) with 1.3 μm central wavelength and 135 nm bandwidth. The axial resolution of the system was 6 μm (in air) and 3.61 μm (in tissue). The transverse resolution of the system was 25 μm. The detector was a 14-bit complementary metal-oxide semiconductor (CMOS) line scan camera (SU-1024LDM Compact; Goodrich, Charlotte, NC, USA) with 1024 pixels. A 50:50 optical fiber coupler was used to split the broadband light beam into the sample and reference arms. A galvanoscanner (GVS002, Thorlabs, Newton, NJ, USA) connected to the sample arm was used to scan the tooth samples. All the samples were scanned with a sufficient cross-sectional scanning range of 1 mm × 1 mm × 1 mm dimensions. A compact spectrometer was designed and contained a collimator, a diffraction grating, an achromatic doublet lens, and a line scan camera. The spectrometer was calibrated to compensate the distortion of the point spread function (PSF), and to improve the signal-to-noise ratio (SNR) up to 110 dB using previous literature reports [28,29]. Further details about the system configuration can be found in Table 1. 

### 2.2. Specimen Preparation

For the proposed preliminary study, four types of *ex vivo* tooth samples, including partially demineralized canine tooth sample, partially demineralized pre-molar tooth sample, partially demineralized molar tooth sample, completely demineralized (carious) molar tooth sample, and *in vivo* healthy molar tooth sample were involved in the experiment. All tooth specimens were examined in patients before extraction. The experimented *ex vivo* tooth specimens were extracted after performing early childhood caries (ECC) surgeries for four orthodontic patients at different age groups (10–12 years) of the dental clinic of the Faculty of Dentistry, Kyungpook National University, Daegu, Korea. The details of the experimented volunteers and tooth specimens are illustrated in Table 2. Prior to the OCT inspection, all *ex vivo* tooth specimens were preserved in sterile filtered de-ionized water solution for 24 h at 30 °C to eliminate any possible superficial enamel cracks and maintain a standard smooth surface after the extraction. The experiments were performed in accordance with the guidelines of the Institutional Animal Care and Use Committee of Kyungpook National University (Daegu, Korea) and approvals from the human ethics committees of the Institute for Bio-diagnostics, Kyungpook National University (Daegu, Korea) were obtained prior to sample collection. 

### 2.3. Intensity Fluctuation Analysis

To evaluate the proposed method precisely, the obtained cross-sectional images were involved in an amplitude scan (A-scan) depth profile analysis to verify the microstructural comparison between healthy, partially demineralized, and carious molar tooth samples. For the A-scan profile analysis, a software-based program was coded using Matlab (Mathworks, Natick, MA, USA) to search the intensity peaks in the depth direction. The acquired 2D OCT image was loaded and a peak search algorithm-based cropped window with 15 intensity signals (A-scan lines) was applied. The developed algorithm detects the maximum intensity in each individual A-scan line to search the peak position, and all the peak positions in all 15 A-scan lines were rearranged while matching the peak intensity index in the A-scans to flatten the region of interest. Owing to the non-flattened region of interest, the maximum intensity index positions vary, and therefore, the index positions with higher intensity values should be rearranged and matched linearly to obtain a flattened image. Finally, all the rearranged and flattened A-scan lines were summed up, averaged, and normalized to obtain a single A-scan depth profile of the region of interest. The applied refractive index of a tooth structure, which affects the depth scale of 2D OCT images was 1.63 [30,31]. Moreover, we performed an additional quantification method to analyze the total intensity fluctuation in deep microstructures according to imaging depth. Thus, an additional automated program was coded using Matlab to analyze the total pixel intensity of each depth range of demodulated 2D OCT images. The analysis was performed for the entire visible depth range of 1 mm, and the total pixel intensity was evaluated for each 250 μm depth range of the cross-sectional image to identify the depth dependent total intensity fluctuation of each tooth specimen category. Then, the entire total pixel intensity of each depth range was summed and averaged for each 2D OCT image. This study was a preliminary observational study, and the data analysis was primarily descriptive. A continuous variation of the optical laser source power was observed, which was compensated afterward. Due to the instability of the laser optical power, the entire intensity of 2D OCT images was compensated by multiplying ±5%. 

### 2.4. Volumetric Analysis

**Figure 2:**
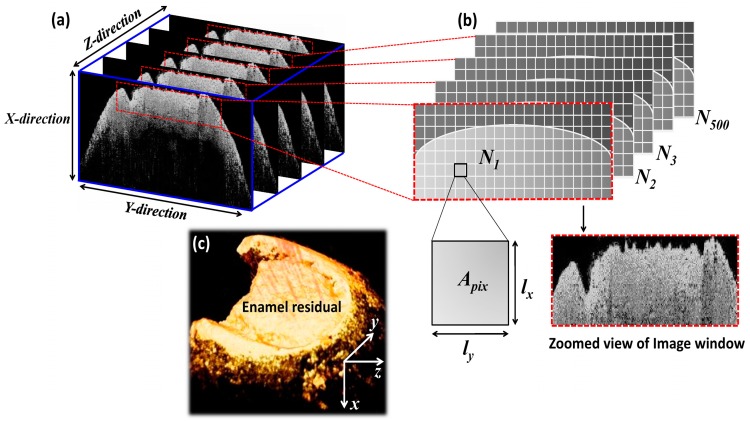
Volumetric evaluation algorithm for enamel residual. (**a**) Sequential 2D OCT images along with the applied image window; (**b**) Detected pixels, which satisfy the applied pre-determined threshold range; (**c**) Acquired 3D OCT volumetric image.

The volumetric measurements of enamel residual was obtained using 2D OCT image based 3D OCT volumetric images by implementing a pixel intensity based automated calculation method, which was performed through a software program coded in Matlab. Figure 2a shows the volumetric calculation algorithm of enamel residual of a single 2D OCT image along with the obtained 3D OCT volumetric image containing 500 2D OCT images (Figure 2c). In the developed algorithm based program, demodulated raw data was loaded and the intensity of the entire cross-sectional region was analyzed. An image window was applied to select the enamel residual region. For the precise selection (filter the region of interest) of the enamel residual region, we approximated the pixel intensity difference between the enamel residual region and other cross-sectional regions, and provided a pre-determined intensity threshold range for a separate evaluation of the enamel residual region. The entire cross-sectional intensity varies from 0 to 255, and the pre-determined enamel threshold (*TH_(en)_*) range can be expressed as, 45 ≤ *TH_(en)_* ≤ 255. The selected threshold range contains the intensity range of the enamel region and excludes the dark black region of the selected image window. The area of a single pixel, which belongs to the selected image window in Figure 2b can be expressed as

(1) $$l_{x}\times l_{y}=A_{pix},$$

where *l_x_ is* the pixel size in the *x*-direction, *l_y_ is* the pixel size in the *y*-direction, and *A_pix_* is the area of a single pixel. The pixel sizes in the *x*-, *y*-, and *z*-directions can be expressed as

$$l_{x}=l_{y}=12\;\mu m,\;\mathrm{and}\;l_{z}=7\;\mu m.$$

The total number of *z*-direction pixels (B-mode images) is 500 owing to the composition of the 3D OCT image. Therefore, the enamel residual volume (volume of the remaining enamel) of 500 2D OCT images (3D volumetric image) can be calculated as

(2) $$\left(N_{1}\times l_{x}\times l_{y}\right)\times l_{z}+\left(N_{2}\times l_{x}\times l_{y}\right)\times l_{z}+\left(N_{3}\times l_{x}\times l_{y}\right)\times l_{z}+\ldots \left(N_{500}\times l_{x}\times l_{y}\right)\times l_{z}=V_{tot},$$

where *N_i_* (*i* = 1, 2, 3, …, 500) is the number of image window pixels in each respective window, which satisfies the pre-determined threshold value range, and *V_tot_* is the evaluated volume of the enamel residual. *N*_1_, *N*_2_, …, *N*_500_ represent the sequential number of respective image window pixels. The accuracy of the developed algorithm can be enhanced by providing a precise pre-determined threshold value range to detect the gradual changes of enamel structure as a result of demineralization.

## 3. Results and Discussion

### 3.1. Morphological Analysis of Dental Caries along with Quantitative Evaluations

**Figure 3:**
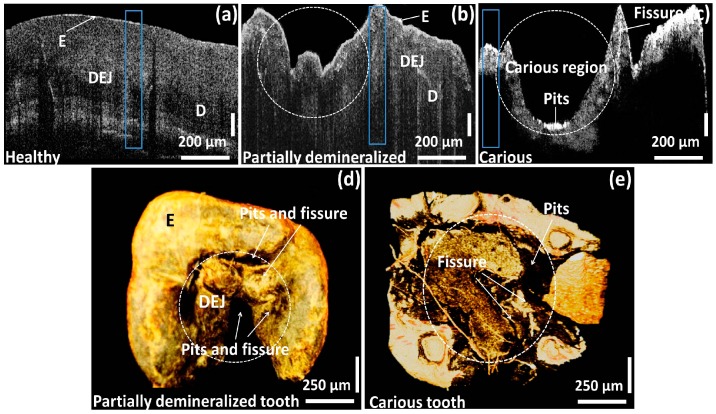
Two-dimensional OCT image comparison between healthy, partially demineralized, and completely demineralized (carious) molar tooth samples along with three-dimensional OCT images. (**a**) 2D OCT image of a healthy molar tooth region; (**b**) 2D OCT image of a partially demineralized molar tooth region; (**c**) 2D OCT image of a completely demineralized (carious) molar tooth region; (**d**) 3D OCT volumetric image of a partially demineralized molar tooth sample; and (**e**) 3D OCT volumetric image of a carious molar tooth sample.

Figure 3a–c show the cross-sectional comparison between healthy, partially demineralized, and completely demineralized (carious) molar tooth samples. Figure 3d,e present the three-dimensional volumetric images of partially demineralized and carious molar tooth samples for a better view. In Figure 3a, the structural layers such as, enamel, dentino-enamel junction, and dentin can be visualized along with the depth ranges of 250 μm, 600 μm, and 800 μm, respectively. The progression of the demineralization can be identified in Figure 3b due to the demineralized enamel region and the formation of pits region in the depth range of 500 μm below the enamel range, and the remaining dentino-junction and dentin layer thickness was about 300 μm. Moreover, a clearly distinguishable demineralized enamel and dentino-junction (in the depth range of 800 μm), which leads to a formation of a carious region including pits and fissures were identified in Figure 3c. In addition, the remaining dentin thickness was about 100 μm. The three-dimensional images (Figure 3d,e) emphasize the top view of the partially demineralized and carious molar teeth samples. The infected microstructures, formation of pits and fissures, as well as the enamel loss, can be clearly visualized through the obtained volumetric images, owing to the high depth penetration. The desired morphological changes owing to demineralization mostly occurred in the enamel and dentin regions, which belong to a depth range that can be sufficiently achieved from the developed OFDI technique, and therefore, the necessity of histological images could be minimized by using the two-dimensional cross-sectional images acquired from the developed non-invasive OCT system [32,33,34,35]. Although the customized SD-OCT system implemented in this preliminary study improves the anatomical evaluation of the enamel and dentin, due to the low image acquisition speed and low sensitivity, the image quality has a limitation in SD-OCT compared to optical frequency domain imaging based high-speed swept source OCT (SS-OCT) [36]. Though the implementation of high-speed 1.3 μm SS-OCT can be beneficial, the customized cost effective OFDI system of this preliminary study was capable of obtaining a sufficient depth visibility to confirm the desired morphological results.

**Figure 4:**
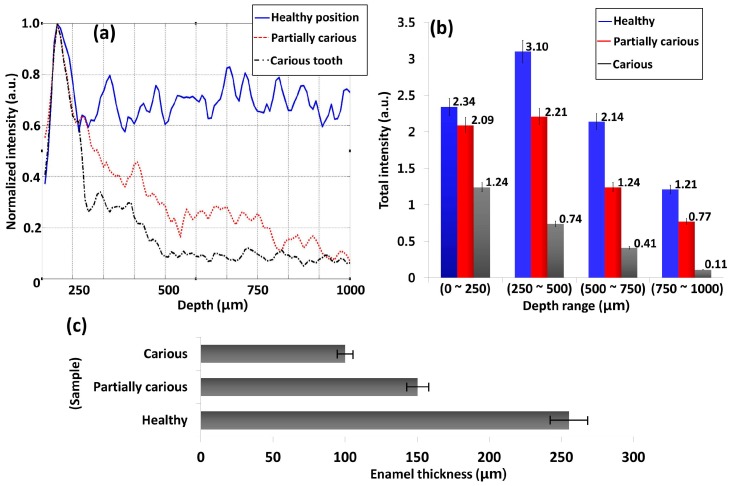
The quantitative evaluation method for healthy, partially demineralized, and completely demineralized (carious) molar tooth samples. (**a**) A-scan depth profiles of healthy, partially demineralized, and carious samples; (**b**) The total intensity fluctuation of healthy, partially demineralized molar, and completely demineralized (carious) molar tooth samples for the entire visible depth range of 1 mm with a gap of 250 μm depth range; (**c**) The depth direction enamel thickness values of the healthy, partially carious, and carious samples.

The blue color box regions of Figure 3a–c depict the averaged A-scan depth profile regions. In Figure 4a, the blue solid line, red dotted line, and black dashed line represent the A-scan depth profiles of healthy, partially demineralized, and carious samples, respectively. Owing to the high scattering coefficient of the healthy structure region, a high backscattered signal intensity can be identified compared to partially demineralized and carious samples. In addition, it is difficult to detect dentin enamel junction and dentin regions of the partially demineralized and carious samples (within depth range of 500 μm to 1000 μm) due to low signal intensity from the structure. The demineralization mainly affects the hard calcium based structural surface of the tooth enamel region by decreasing the calcium constitution and simultaneously increase the progression of caries, which contain soft tissues and blood vessels. Owing to the aforementioned increase of the soft tissue region containing blood vessels, light scattering coefficient decreases. Therefore, the degree of demineralization affects the OCT signal in depth direction. As a consequence, low signal intensity could be detected from partially demineralized samples compared to healthy samples.

The aforementioned depth dependent intensity fluctuation according to each sample within each depth range is shown in the Figure 4b graph. The total intensity fluctuation of the healthy sample, partially demineralized sample, and carious sample are illustrated in blue, red, and gray color plots, respectively. Owing to the high optical scattering coefficient, the healthy sample performs the highest total intensity in all depth ranges, and the least intensity values could be identified in the carious sample. Furthermore, the partially demineralized sample performed less total intensity than the healthy sample and a higher intensity than the carious sample, which confirms the progression of dental caries. All the obtained intensity values are shown along with the corresponding graph bars. Furthermore, the enamel thickness was evaluated, and the next bar graph shown in Figure 4c represents the depth direction enamel thickness values of the aforementioned three samples using two-dimensional cross-sectional image based A-scan depth profile analysis. The thickness of the healthy enamel region was measured as 255.45 ± 15.03 μm, a partially demineralized molar specimen was measured as 150.30 ± 10.02 μm (with a reduction of 41.1% compared to the healthy sample), and the enamel residual of the carious region was measured as 100.20 ± 6.68 μm (with a reduction of 60.8% compared to the healthy sample), respectively. 

**Figure 5:**
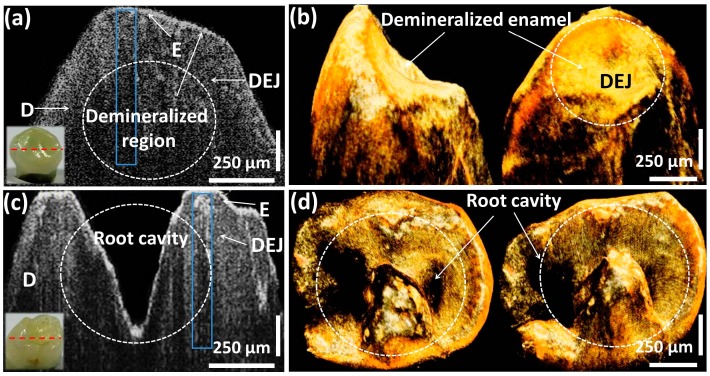
Two-dimensional OCT image comparison between a partially demineralized but healthy appearing canine tooth sample and a partially demineralized but healthy appearing pre-molar tooth sample with three-dimensional OCT images. (**a**) 2D OCT image of a partially demineralized but healthy appearing canine tooth sample; (**b**) 3D OCT volumetric image of a partially demineralized but healthy appearing canine tooth sample; (**c**) 2D OCT image of a partially demineralized but healthy appearing pre-molar tooth sample; (**d**) 3D OCT volumetric image of a partially demineralized but healthy appearing pre-molar tooth sample.

To gain a better understanding about the enamel demineralization, we repeated the aforementioned similar experimental method using partially demineralized canine and pre-molar tooth samples, which have a healthy appearance externally. Figure 5a,c emphasize the 2D OCT images along with the acquired 3D OCT volumetric images (Figure 5b,d) and sample photographs. The scanned positions of the samples are shown by red dashed lines. Although the samples have a healthy appearance, the formation of the demineralized enamel and root cavity region were identified in cross-sectional images and 3D OCT images of both canine and pre-molar tooth samples. Moreover, the enamel of the pre-molar tooth sample was demineralized towards a 750 μm depth to form the cavity region, which can be quantitatively evaluated using A-scan depth profile analysis. To acquire the most accurate quantification of enamel residual and the demineralized enamel region, we repeated the previous quantitative methods under the same experimental conditions.

**Figure 6:**
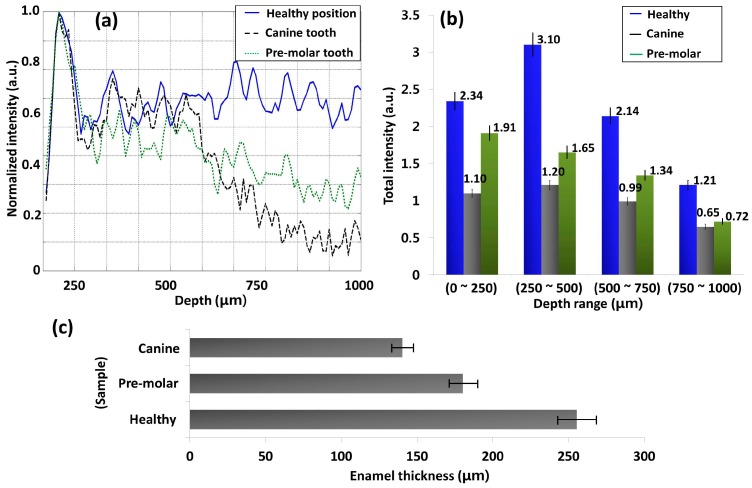
The quantitative evaluation for a healthy sample and partially demineralized but healthy appearing canine and pre-molar tooth samples. (**a**) A-scan depth profiles of healthy and partially demineralized but healthy appearing canine and pre-molar tooth samples; (**b**) the total intensity fluctuation of healthy, canine, and pre-molar tooth samples for the entire visible depth range of 1 mm with a gap of 250 μm depth range; (**c**) the depth direction enamel thickness values of the healthy, partially demineralized but healthy appearing canine and pre-molar tooth samples.

The obtained A-scans of partially demineralized canine tooth sample and partially demineralized pre-molar tooth sample were compared with a healthy sample and shown in Figure 6a. In the A-scan profile, the blue solid line, black dashed line, and green dotted line represent the A-scan depth profiles of healthy sample, canine sample, and pre-molar sample, respectively. Similar signal intensity behavior was confirmed as before, owing to the low scattering coefficient of partially demineralized samples, which confirms the changes of the sample composition compared to the healthy sample. Then, we analyzed and compared the total intensity fluctuation of both partially demineralized samples according to each imaging depth range (Figure 6b). 

All the obtained intensity values are shown along with the corresponding graph bars. Although a reduction of the total intensity was noticed in partially demineralized samples (compared to the healthy sample), all the intensity levels were comparatively higher than the intensity levels of the carious sample. Therefore, the results confirm that the experimentally tested samples were neither healthy nor carious and verify the progression of demineralization. Next, we analyzed and compared enamel thickness of all three samples, and the bar graph shown in Figure 6c represents the obtained depth direction enamel thickness. The thickness of the healthy enamel region was measured as 255.45 ± 15.03 μm, partially demineralized pre-molar tooth sample was measured as 180.36 ± 12.02 μm (reduction of 29.4% compared to the healthy sample), and partially demineralized canine tooth sample was measured as 140.28 ± 9.35 μm (reduction of 45.1% compared to the healthy sample), respectively. Therefore, the obtained quantitative evaluations confirm the progression of early caries, and, moreover, the obtained quantitative evaluations can be utilized as threshold parameters to detect the progression of early caries. To gain a better understanding about the quantified thickness values and total intensity fluctuations of the experimented tooth specimens, the summarized quantifications are illustrated in Table 3.

### 3.2. Volumetric Evaluation Technique to Identify Initial Caries

We quantified the volume of the enamel residual by applying the described volumetric algorithm in Section 2.4. Thus, we calculated the enamel residual of a selected particular position determined by the expert orthodontist for the healthy tooth specimen, three partially demineralized but healthy appearing tooth specimens, and the carious tooth specimen. All the performed calculations were based on the refractive index of 1.63, and the calculated enamel residual volume evaluations are illustrated along with the parameters in Table 4. 

Hence, the obtained results confirmed that the proposed volumetric evaluation method will be more useful to detect the progression of caries, since the gradual reduction of the enamel volume owing to the gradual growth of caries can be detected quantitatively in advance. Therefore, medical treatments can be initiated immediately in order to obstruct the progression of caries, once the volume reduction of teeth is identified. 

### 3.3. Structural Comparison between OCT and Conventional Methods

**Figure 7:**
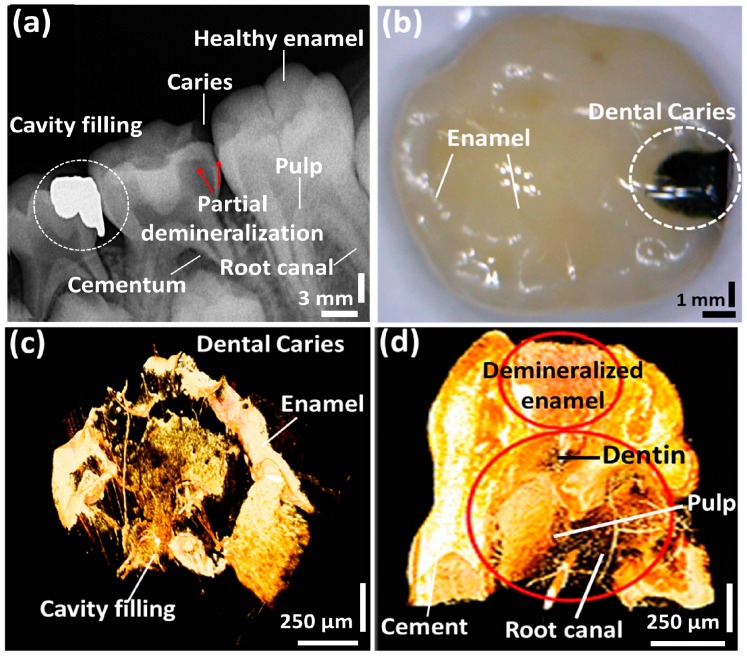
Structural analysis of the carious tooth sample using various inspection methods. (**a**) Radiographic image of the carious tooth sample before the surgery; (**b**) Photograph of the carious tooth sample after the surgery; (**c**,**d**) top and side views of 3D OCT images of the carious tooth sample.

Figure 7 shows the structural analysis of a carious molar tooth sample and a comparison between imaging results obtained from various inspection methods. Figure 7a shows the *in vivo* radiographic image of the carious tooth, which was captured before early childhood caries (ECC) surgery performed on a 10-year-old male volunteer. The images were acquired to inspect the dental caries and cavity filling portions using a system that is currently applied in standard clinical practice: ultra speed (D-speed) film (Kodak, Rochester, NY, USA), 150 kVp, 15 mA, and 20 impulses. In this radiographic method, resolving a sub-millimeter tissue structure proves to be difficult, and only the surface structures of the cavity fillings, carious region, partially demineralized regions, pulps, and root canals along with healthy tooth were visualized. Thus, the obtained results were neither quantitative nor sensitive, and the cavity depths could not be imaged as well. Hence, precise radiographic detection of demineralization is a challenging task, since minimally demineralized regions are unable to reach the threshold of resolution. Figure 7b shows the photograph of the carious tooth, which was obtained after early childhood caries (ECC) surgery. Figure 7c,d represent the *ex vivo* 3D OCT images of the same sample and show the top and the side views of the sample. A precise enhancement could be identified, compared to radiographic images. Both 3D OCT figures give a clear view of the distinguishable anatomical structures e.g., dentin tubules, pulp, root canals, and cement owing to the high axial and lateral resolutions. Therefore, the applicability and the reliability of our system were verified because the dental caries, demineralization, and the inner microstructures of dentin were confirmed simultaneously through the obtained results.

## 4. Conclusions

We have demonstrated an optical frequency-domain imaging technique based quantitative evaluation method as an initial *ex vivo* study to detect the progression of dental caries by comparing partially and completely demineralized tooth samples with healthy tooth specimens. The quantification techniques were carried out by evaluating precise volume and thickness of enamel residual. Next, the total intensity fluctuation in each imaging depth range of all the specimens was quantified to confirm the changes that occurred in the internal composition of partially and completely demineralized tooth samples compared to a healthy sample. The performed study was a preliminary descriptive observational study, which was performed to confirm the feasibility of the three developed quantification techniques. The representative *ex vivo* tooth specimens as well as the experimental procedure was conducted according to the guidelines provided by an expert orthodontist. The results obtained using our high-resolution OFDI system revealed anatomic and quantitative information in a relatively nondestructive manner. The threshold parameters to detect the progression of early caries were determined on the basis of the quantitative results obtained from partially demineralized samples. Therefore, the physicians were able to diagnose the tooth volumetric and thickness changes at an initial stage by considering the obtained results as promising threshold parameters, which will be useful to barricade the progression of caries. To enhance the accuracy of the threshold parameters, quantitative (thickness and volumetric) information of multiple *in vivo* specimens will be evaluated, averaged, and normalized along with clinical trials in future studies.

## Figures and Tables

**Table 1:** The details of the optical frequency domain imaging system.

System Parameters	Specification
Central Wavelength	1310 nm
Spectral bandwidth	135 nm
Axial resolution air/tissue	6 μm/3.61 μm
Transverse resolution	25 μm
Maximum imaging width	8 mm
Maximum imaging depth	>6 mm
Optical power variation	±5%

**Table 2:** The details of the experimented tooth specimens.

Experimented Volunteer	Tooth Classification	Inspection Category
11-year-old male	Molar tooth	Healthy
11-year-old female	Molar tooth	Partially demineralized
10-year-old male	Molar tooth	Carious
11-year-old male	Canine tooth	Partially demineralized
12-year-old female	Pre-molar tooth	Partially demineralized

**Table 3:** The quantified enamel thickness and depth direction total intensity fluctuations.

Specimen Category	Enamel Thickness (μm)	Total Intensity Fluctuation in Each Depth Range (a.u.)			
		0–250 μm	250–500 μm	500–750 μm	750–1000 μm
Healthy molar	255.45 ± 15.03	2.34 ± 0.2	3.10 ± 0.2	2.14 ± 0.2	1.21 ± 0.2
Dem. molar	150.30 ± 10.02	2.09 ± 0.2	2.21 ± 0.2	1.24 ± 0.2	0.77 ± 0.1
Carious molar	100.20 ± 6.68	1.24 ± 0.1	0.74 ± 0.05	0.41 ± 0.02	0.11 ± 0.01
Dem. canine	140.28 ± 9.35	1.10 ± 0.1	1.21 ± 0.1	0.99 ± 0.1	0.65 ± 0.1
Dem. premolar	180.36 ± 12.02	1.91 ± 0.1	1.65 ± 0.1	1.34 ± 0.1	0.72 ± 0.05

**Table 4:** The volumetric evaluation results of the enamel residual.

Tooth Specimen	Total Number of 2D OCT Images	Total Number of Enamel Residual Pixels	Enamel Residual Volume (mm^3^)
Healthy molar tooth	500	2.13 × 10^7^	28.72
Part.dem. molar	500	1.31 × 10^7^	17.70
Carious molar	500	0.91 × 10^7^	12.26
Part.dem. canine	500	1.28 × 10^7^	17.20
Part.dem. pre-molar	500	1.42 × 10^7^	19.15
